# Is self-reported park proximity associated with perceived social disorder? Findings from eleven cities in Latin America

**DOI:** 10.1016/j.landurbplan.2021.104320

**Published:** 2022-03

**Authors:** Mika R. Moran, Daniel A. Rodríguez, Andrea Cortinez-O'ryan, J. Jaime Miranda

**Affiliations:** a Institute for Urban and Regional Development, University of California Berkeley, Berkeley, CA 94720, USA; b Department of City and Regional Planning, University of California Berkeley, Berkeley, CA 94720, USA; c Departamento de Educación Física, Deportes y Recreación, Universidad de la Frontera, Temuco, Chile; d CRONICAS Centre of Excellence in Chronic Diseases, Universidad Peruana Cayetano Heredia, Lima, Peru; e Department of Medicine, School of Medicine, Universidad Peruana Cayetano Heredia, Lima, Peru

**Keywords:** Parks, Crime, Built environment, Latin America, Informal neighborhoods, Social disorder, Equity

## Abstract

Parks and greenspaces can enhance personal health in various ways, including among others, through psychological restoration and improved well-being. However, under certain circumstances, parks may also have adverse effects by providing isolated and hidden spaces for non-normative and crime-related activities. This study uses a survey conducted by the Development Bank of Latin America in a cross-sectional representative sample of 7,110 respondents in eleven Latin-American cities. We examine associations between self-reported park proximity with perceived social disorder (drug use/sales, gangs, prostitution and assault and/or crime), and whether these associations are modified by neighborhood characteristics (informal neighborhoods, poor street-lighting, abandoned buildings, illegal dumping). High self-reported park proximity was associated with lower perceptions of social disorder, but these associations were no longer significant following adjustment for neighborhood characteristics. Significant interactions were observed between park proximity and neighborhood characteristics suggesting that the likelihood of perceiving social disorder increases with high park proximity in informal neighborhoods and in the presence of certain neighborhood characteristics, such as poor street-lighting, abandoned buildings, and illegal dumping in residential streets. The differential associations between reported park proximity and perceived social disorder in different living environments highlight the importance of supportive social and physical infrastructure to maximize the restorative benefits of parks in all urban areas.

## Highlights

• Social disorder is higher in informal neighborhoods and where street infrastructure is poor.
• Park proximity is associated with less/more social disorder in formal/informal neighborhoods.
• Park proximity is associated with less social disorder in streets with good infrastructure.
• Park proximity is associated with more social disorder in streets with poor infrastructure.
• Investment in neighborhood/street infrastructure can maximize their restorative benefits of parks.

## 1 Introduction

Parks can contribute to individual and community safety and well-being by providing opportunities for psychological restoration, physical activity and social interactions (Maas et al., 2009, Markevych et al., 2017). The mere exposure to green space (having it nearby, looking at it) is known to have restorative benefits through stress relief and the reduction of anger and aggression, which can then ultimately reduce anti-social behaviors (Kuo and Sullivan, 2001a, Kuo and Sullivan, 2001b). At the same time, parks and green spaces provide opportunities to engage in physical activity and enhance social interactions (Bedimo-Rung et al., 2005), which consequently improve personal safety by increasing surveillance (Kuo and Sullivan, 2001a, Kuo and Sullivan, 2001b) while potentially enhancing social capital (Markevych et al., 2017).

Of these various influences, the impact of parks on social disorder gained research interest in recent years along with the increasing recognition of social disorder as an important public health issue (Ndjila et al., 2019). As a construct, social disorder has various definitions and typologies (Bogar and Beyer, 2016, Ndjila et al., 2019), including physical (e.g., graffiti, vandalism) and social elements (e.g., violence, gangs, drug use). This paper focuses on residents’ perceptions of the social elements of disorder.

Social elements of disorder have been assessed in previous studies both subjectively through surveys (Evenson et al., 2012), and objectively through direct observations documenting violence, gangs and drug activities (Cohen et al., 2016), as well as based on police reports of assault (Boessen and Hipp, 2018, Kamal and Suk, 2018), prostitution (Evenson et al., 2012), gangs and drugs (Evenson et al., 2012, Kamal and Suk, 2018). Social disorder has detrimental effects on various mental and physical health conditions, such as depression (Nowak et al., 2020), cognitive decline (Boardman et al., 2012), diabetes (Steve et al., 2016), cardiovascular diseases (Barber et al., 2016) and unintentional injuries (Moreira et al., 2020). Both perceived and objective social disorder measures were found to be associated with park access (Bogar & Beyer, 2016), park use (Bedimo-Rung et al., 2005, Leslie et al., 2010, Stodolska et al., 2009), and related health benefits, such as outdoors walking and physical activity (Evenson et al., 2012), and mental health (Leslie & Cerin, 2008).

Despite accumulating evidence associating parks with increased safety and reduced social disorder (Bedimo-Rung et al., 2005, Kuo and Sullivan, 2001a, Kuo and Sullivan, 2001b, Markevych et al., 2017), this may not always be the case. Urban parks, typically owned and managed by the city and open to the public, belong to everyone and to no one at the same time. Thus, under certain circumstances, urban parks may decrease personal safety by, for example, providing isolated and occluded spaces that allow non-normative and crime-related activities. This is especially likely in parks with no lighting that remain open after dark and lack surveillance. On the other hand, parks with good lighting and/or those that are closed to the public after dark are less likely to attract social disorder. In some case, parks that attract many visitors may also attract social disorder behaviors. For example, in Los Angeles (California), more park visitors were documented in parks that had more gangs and intimidating groups in conflict (Cohen et al., 2016). However, this could be due to the mere fact that large parks, or parks at central and accessible locations, can attract both normative and non-normative populations. Previous studies largely report negative associations between park use with social disorder (Sreetheran & van den Bosch, 2014), but other evidence also exists showing positive (Moran et al., 2020, Ribeiro et al., 2015) or null associations (Moran et al., 2020). In line with these inconsistencies, a recent review of US studies (Bogar & Beyer, 2016) showed differential associations between parks and different types of social disorder by linking parks with less violence crime (e.g., assault, manslaughter), but more property crime (e.g., burglaries, car theft) and nuisance crime (e.g., narcotics sales and possession, public drunkenness). However, these inconsistent findings are likely attributed to the use different measures of disorder in different studies and thus should be interpreted with caution. This duality in which parks can serve as both crime inhibitors and facilitators (Boessen & Hipp, 2018) is also likely to affect park’s ability to support physical and mental health. It is therefore important to work towards a nuanced understanding of associations between parks and social disorder, which can then ultimately help develop context-sensitive park policies to address community safety and well-being.

The impact of such contextual factors on social disorder was recognized and articulated in prominent urban design approaches. Essentially, the multidisciplinary approach of “crime-prevention through environmental design”, incorporates architecture, urban design, and psychology to develop design principles to deter anti-social and criminal behaviors (Jacobs, 2016, Newman, 1996). One principle, for example, concerns limiting overgrown vegetation as a mean to increase visibility, as parks with overgrown vegetation make both offenders and potential victims less visible, and thereby may increase the likelihood of attacks (Jansson et al., 2013). Another principle is street-lighting, which attempts to increase visibility and enable natural/informal surveillance (e.g., by residents or by-passers), especially after dark. A UK study further found that street-lighting can reduce both crime rates and crime related financial costs (Painter & Farrington, 2001). These ideas, are reinforced by Jane Jacobs’ (Jacobs, 2016) concept of “eyes on the street”, suggesting that natural surveillance provided casually by locals within the communities can be a cost-effective substitute, and even outperform official policing in increasing social control. While these principles were originally suggested by professionals, they are often echoed in empirical studies that investigated the perspectives of park users (Jansson et al., 2013, Sreetheran and van den Bosch, 2014).

The varying effects of parks on social disorder were attributed to characteristics of parks and their surroundings. High quality and well-maintained parks with supportive facilities and amenities are likely to increase safety, while those with low-quality and poorly maintained infrastructure are likely to be neglected and vandalized (Bedimo-Rung et al., 2005). Park vegetation is also influential. Parks with low-density, open vegetation are more likely to be perceived safe compared to parks with poorly maintained, dense, and enclosed vegetation (Jansson et al., 2013). In the Netherlands, green spaces were found to enhance perceived safety in urban and rural areas, with the exception of highly dense urban areas, where enclosed green spaces were associated with a reduced sense of safety (Maas et al., 2009). Similarly, parks located in affluent neighborhoods were perceived by residents as enhancing personal safety, while those in deprived areas were likely to be underutilized and perceived by residents as more vulnerable for neglect and crime (Leslie et al., 2010, Wilson et al., 2004). Another important contextual attribute that is likely to influence perceived safety is parks’ spillover effects – the process by which park use and related activities may extend beyond parks into their surroundings (Crewe, 2001). In a recent U.S. study, Boessen and Hipp (2018) found that land uses and socio-demographic characteristics nearby parks are related to crime reports both within and nearby parks.

The importance of context raises the prospect that neighborhood characteristics may also interact with park characteristics in influencing perceived and actual safety. For example, park amenities, visibility, and vegetation may influence actual and perceived safety differently depending on where the park is situated. Indeed, a few recent studies in high income contexts have examined such interactions (Lo and Jim, 2010, Maas et al., 2006). According to these studies’ results, the presence of certain types of green space was more strongly related to residents’ concerns about safety in parks located at the urban core (Lo & Jim, 2010) and in areas with higher levels of urban density (Maas et al., 2009). Whether similar interactions exist in middle income countries, where urban context variations are likely to be more pronounced, remains to be determined. To address this, the current study investigated the potential impact of self-reported park proximity on perceived social disorder conditions, and how this impact may vary by neighborhood characteristics in Latin America – a less studied yet highly vulnerable world region suffering from a paucity of urban green space (Rigolon et al., 2018) and high crime rates (Ayres, 1998), along with rapid urbanization and high social inequalities across and within cities (Vereinte Nationen, 2013).

Research questions

***Research Question 1:*** Is self-reported park proximity associated with perceived social disorder around home?

***Hypothesis 1:*** This question is exploratory. We do not have a clear expectation regarding the direction of this association, as suggested by the multiple possible causal pathways and inconsistent findings in the extant literature.

***Research Question 2:*** Are neighborhood characteristics associated with perceived social disorder around home?

***Hypothesis 2:*** Participants are more likely to report social disorder if they live in informal neighborhoods or in areas with unpaved streets, poor street-lighting, abandoned buildings, or illegal dumping.

***Research Question 3:*** Do associations between self-reported park proximity and perceived social disorder conditions around home vary by neighborhood characteristics?

***Hypothesis 3:*** We expect higher park proximity to be associated with fewer reports of social disorder for participants living in formal neighborhoods or in areas with any of the following charachteristics: Sidewalks, paved streets, good street-lighting, no abandoned buildings and illegal dumping. Conversely, we expect higher park proximity to be associated with more reports of social disorder for participants living in informal neighborhoods or in areas with any of the following charachteristics: no sidewalks, unpaved streets, poor street-lighting, abandoned building, and illegal dumping.

## 2 Materials and methods

### 2.1 Participants and procedures

Data for this study come from a cross-sectional, stratified, representative survey conducted by the Development Bank of Latin America (henceforth: CAF survey). The survey questionnaire (CAF, 2016) includes modules on urban transportation, safety, garbage collection, water and sanitation, electric energy, and housing. Data collection was carried out between November 2016 through January 2017 by interviewing one adult (aged 20–60) per household. The CAF survey used a probabilistic sampling of urban blocks with probability proportional to size (usually block population), stratified by geographical areas and/or socioeconomic level of the block, depending on the city. After the blocks were selected, households were systematic selected with quotas by age and sex groups. A detailed description of the sampling method is described elsewhere (CAF, 2016).

Overall, 12,905 participants responded to the CAF survey from the following 11 Latin American cities: Buenos Aires (Argentina), Bogota (Colombia), Caracas (Venezuela), Fortaleza (Brazil), La Paz (Bolivia), Lima (Peru), Mexico City (Mexico), Montevideo (Uruguay), Panama City (Panama), Quito (Ecuador) and Sao Paulo (Brazil). The sample from the latter four cities included also informal neighborhoods, defined as a set of more than 50 contiguous dwellings with (1) no property title, (2) building deficiencies, and (3) lack of formal access to public services of water, electricity and sanitation. Of the 12,905 individuals who participated in the CAF survey, 7,110 participants were included in this analysis. 961 individuals were excluded because they did not have complete information on social disorder, 446 did not have complete information on street environment, and 4,388 did not have all individual socio-demographic characteristics. The original CAF survey sample (N = 12,905) did not differ significantly in measured sociodemographic characteristics from the one used for our analysis (n = 7,110).

### 2.2 Measures and variables

Perceived social disorder is the study outcome as defined by different types of non-normative and anti-social behavior described below (section 2.2.1). Self-reported park proximity is the main exposure, neighborhood characteristics (objective and self-reported) are potential moderators, and individual characteristics are controls variables.

#### 2.2.1 Outcomes

*Perceived social disorder:* four perceived social disorder outcomes were included reflecting the presence of any of the following conditions nearby the respondent’s home: *Drug use or sale*, *gangs*, *prostitution* and *assault or crime*. *Drug use or sale* was assessed by a single question asking participants whether “spending on drug use” occur within three blocks or less from their home (0 = no, 1 = yes). These three other social disorders – *gangs*, *prostitution* and *assault or crime –* were assessed by a single question in the CAF survey instrument asking participants to assess how often each of these situations occurs in their residential block by using a scale of 1–5 when: 1 = “never”, 2 = “rarely”, 3 = “sometimes”, 4 = “almost always”, 5 = “always”. The answers were then recoded to create a binary variable when 0 = “never or rarely” and 1 = “at least sometimes”. To assess overall disorder, we combined the four social disorder domains into a summary binary variable distinguishing between participants who reported at least one type of social disorder from those who did not report any social disorder. This summary variable was calculated in two steps: (1) We summed the four social disorder variables to create an ordinal variable ranging from 0 to 4, where the sum reflects the number of social disorders reported; and (2) This ordinal variable was then recoded into a binary variable distinguishing between participants who “reported one or more social disorder domains” (coded as “1”) and those who “did not report any social disorder” (coded as “0”).

#### 2.2.2 Exposures and effect modifiers

*High park proximity (exposure):* Participants were asked how long would it take them to walk from their home to the nearest “park, square or green space” by selecting one of three options: “less than 10 min”, “10-30 min” and “more than 30 min”. Based on prior studies (Saelens et al., 2002, Saelens et al., 2003), we recoded respondents answers to a binary variable reflecting high park proximity, in which 1 = “having a park in less than 10 min walking from home”, and 0 = “having a park more than 10 min walking from home”.

*Neighborhood and street characteristics (exposures and effect modifiers):* The following five neighborhood characteristics were examined, including predefined surveyors' observations and self-reported measures.

*Neighborhood characteristics:*

*(1) Neighborhood type* – neighborhood type was predefined through the sampling process and was included in the analysis as a binary variable where 0 = formal neighborhood, 1 = informal neighborhood.
*(2) Neighborhood infrastructure*:
*(2a) Unpaved streets –* surveyors reported the type of the street where participants live by selecting one of four categories: “paved street”, “alleyway”, “unpaved” or “other”. For analysis purposes, this was recoded as 1 = “paved street” and 0 = “other/non-paved street”.
*(2b) Lack of sidewalks* – surveyors reported whether participants had sidewalks in the street where they live or not. This variable was coded as; 0 = “Sidewalks present”, 1 = “sidewalks absent”.
*(2c) Poor street-lighting* – participants were asked whether they had “poorly lit street” within three blocks from their home. For analysis purposes, the participants’ answers were inverted to create a variable representing proper street-lighting (1 = “yes”, 0 = “no”).
*(2d) Abandoned buildings –* participants were asked whether they have abandoned buildings within three blocks or less from their home; the answers were coded as: 0 = no, 1 = yes.
*(2e) Illegal dumping –* participants were asked whether they have illegal dumping within three blocks or less from their home; the answers were coded as: 0 = no, 1 = yes.

*Individual characteristics (control variables):* Demographic variables included as controls were: Sex, age, having school aged children (aged 4–18) (yes/no), length of residence in the neighborhood (in years), and self-rated health (good/regular/bad). Socioeconomic indicators, such as vehicle ownership, employment status and education, were also included. Vehicle ownership was defined as: 0 = “there are no cars in the household” and 1=“there is at least one car in the household”. The CAF survey included multiple employment categories, which were reduced for analysis purpose to yield a binary variable where 0 = “unemployed” and 1 = “employed”. Education also included multiple categories which were similarly reduced to create a binary variable where 0 = “having less than high-school education” and 1 = “having high-school education or higher”. Household overcrowding was assessed as the floor area per person in household. In addition, we adjusted for park use (1 = “yes”, 0 = no”) by using the question: “Do you or another member of your household visit parks, squares or green areas on a regular basis?”.

### 2.3 Analysis

Conventional summary statistics were used to describe the sample. To test the first and second research questions, multilevel logistic regression models were estimated for each of the five social disorder outcomes (overall disorder, drug use or sale, gangs, prostitution, and assault or crime) with random effect at the city level to account for heterogeneity across cities. High park proximity (<10 min) and the six neighborhood characteristics variables were entered separately into each of the five models while controlling for individual characteristics. This resulted in 35 partially adjusted associations (Table 2 – in which each cell represents associations estimated in one model). In addition, park proximity and neighborhood characteristics with p-value < 0.05 in the partially adjusted models were then included simultaneously in fully adjusted models, one per social disorder outcome (Appendix 1 – in which each column represents associations estimated in one model).

**Fig. 1:**
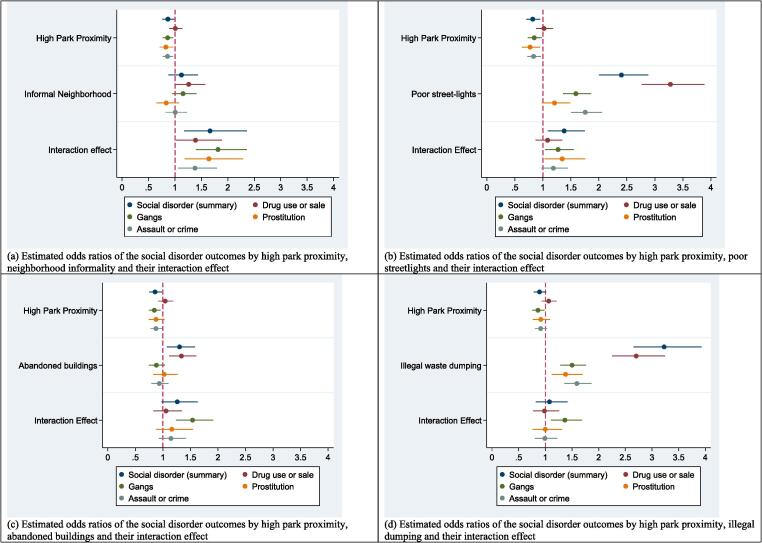
Estimated odds ratios of social disorder outcomes by high park proximity, neighborhood characteristics and their interaction effect (n = 7110).

To addresses effect modification (research question 3), we created interaction terms between high park proximity and four neighborhood characteristics with p-value < 0.05 in the partially adjusted models. For each social disorder outcome, four separate regression models were estimated, one for each block of park proximity, neighborhood characteristic, and their interaction variables, while adjusting for individual characteristics. Fig. 1 and Appendix 2 show estimated odds ratios for the main and interaction effects of high park proximity and neighborhood characteristics.

For all outcomes, multilevel logistic regression models with city-specific random effects were estimated. To account for the large number of models, results were further tested using the Benjamini-Hochberg procedure (Mcdonald, 2009) with a false-discovery rate of 10%.

Estimates are accompanied by 95% CIs and a p-value < 0.05 was used to identify statistical significance when interpreting model results. Statistical analyses were conducted using Stata v15 (Stata Corporation, College Station, TX). Regression models (Table 2 and Appendices 1 and 2) were implemented using the *meqrlogit* Stata command, and coefficient plots (Fig. 1) were obtained using the *coefplot* Stata command.

We had concerns about residential self-select bias suggesting that individuals select neighborhoods based on their personal characteristics (e.g., income, fear of crime) and thus, in this study, observed associations between neighborhood characteristics and social disorder may be an artifact caused by this residential self-selection process. To address these concerns, we conducted a sensitivity analysis repeating all the models after excluding from the sample 180 participants who reported choosing their neighborhood because it had “safety and low crime” (n = 137) and high “proximity to parks and squares” (n = 43). The results remained essentially the same, suggesting that residential self-selection is not likely to affect our analysis. In another sensitivity analysis, we repeated all the analyses on a subsample of the four cities that included informal neighborhoods (with a total of 3,316 residents). The results (not reported) remained consistent with those presented here (Table 2 and Appendices 1 and 2) for the sample of 11 cities and 7,110 residents.

## 3 Results

### 3.1 Sample characteristics

Table 1 contains descriptive statistics of the study variables in the total sample and by the overall disorder (one or more reported vs. none reported). Most of the sample (71%) reported having at least one type of social disorder in their residential street. Of the four social disorder outcomes, drug use/sale was most reported (57%) followed gangs (44%) and assault or crime (49%, Table 1). Prostitution, however, was reported by only 16% of the participants. Social disorder facets slightly varied across cities. In most cities, except La Paz and Panama City, drug use/sale was reported by over 52% of participants. Presence of gangs and assault/crime was reported by over 40% of the residents in eight out of the 11 cities. Prostitution was the least frequently reported social disorder across cities and ranged from 5% to 40%.

**Table 1:** Sample characteristics (count, percent).

Variables		Total sample (n = 7110)	Social disorder summary	
			None reported (n = 2029)	1 or more reported (n = 5081)
*social disorder domains*	Social disorder summary (1 or more reported)	5,081 (71%)	0 (0%)	5,081 (100%)
	Drug use/sales	4,032 (57%)	0 (0%)	4,032 (100%)
	Gangs	3,142 (44%)	0 (0%)	3,142 (100%)
	Prostitution	1,152 (16%)	0 (0%)	1,152 (100%)
	Assault or crime	3,461 (49%)	0 (0%)	3,461 (100%)

*Park proximity*	Less than 10 min' walk	3,867 (54%)	1,161 (30%)	2,706 (70%)
	More than 10 min’ walk	3,243 (46%)	868 (27%)	2,375 (73%)

*Neighborhood characteristics*				
*Neighborhood type*	Informal	1,294 (18%)	207 (16%)	1,087 (84%)
	Formal	5,816 (82%)	1,822 (31%)	3,994 (69%)

*Street characteristics*				
*Street pavement*	House on other street type (i.e., dirt, alleyway, other)	1,471 (21%)	413 (28%)	1,058 (72%)
	House on paved street	5,639 (79%)	1,616 (29%)	4,023 (71%)

*Sidewalk*	No sidewalks in residential street (within block)	2,242 (32%)	691 (31%)	1,551 (69%)
	Sidewalks are present in residential street (within block)	4,868 (68%)	1,338 (27%)	3,530 (73%)

*Street-lighting*	Poor street-lighting within three blocks	3,501 (49%)	586 (17%)	2,915 (83%)
	Good street-lighting within three blocks	3,609 (51%)	1,443 (40%)	2,166 (60%)

*Abandoned building*	There are abandoned buildings within three blocks	2,112 (30%)	433 (21%)	1,679 (79%)
	There are no abandoned buildings within three blocks	4,998 (70%)	1,596 (32%)	3,402 (68%)

*Waste dumping*	There is illegal dumping within three blocks	2,340 (33%)	337 (14%)	2,003 (86%)
	There is no illegal dumping within three blocks	4,770 (67%)	1,692 (35%)	3,078 (65%)

*Individual characteristics*				
*Sex*	Male	3,038 (43%)	937 (31%)	2,101 (69%)
	Female	4,072 (57%)	1,092 (27%)	2,980 (73%)
	Age [M(SD)]*	40.10 (0.14)	40.29 (0.25)	40.02 (0.16)
	Length of neighborhood residency in years [M(SD)]*	20.30 (0.18)	19.35 (0.35)	20.67 (0.21)

*School aged children*	Have school aged children	4,604 (65%)	1,261 (27%)	3,343 (73%)
	Does not have school aged children	2,506 (35%)	768 (31%)	1,738 (69%)

*Parks use*	Park user	4,690 (66%)	1,378 (29%)	3,312 (71%)
	Non-park user	2,420 (34%)	651 (27%)	1,769 (73%)

*Automobile ownership*	Automobile owner	2,235 (31%)	725 (32%)	1,510 (68%)
	Non-automobile owner	4,875 (69%)	1,304 (27%)	3,571 (73%)

*Employment status*	Employed	4,548 (64%)	1,327 (29%)	3,221 (71%)
	Unemployed	2,562 (36%)	702 (27%)	1,860 (73%)
	Overcrowding – Area per person in the household (m^2^) [M(SD)]	23.88 (0.23)	25.88 (0.45)	23.08 (0.27)

*Education*	Less than high school	3,270 (46%)	797 (24%)	2,473 (76%)
	High school or higher	3,840 (54%)	1,232 (32%)	2,608 (68%)

*Self-rate health*	Bad	231 (3%)	43 (19%)	188 (81%)
	Regular	2,274 (32%)	599 (26%)	1,675 (74%)
	Good	4,605 (65%)	1,387 (30%)	3,218 (70%)

*City of residence*				
	Buenos Aires	972 (14%)	226 (23%)	746 (77%)
	La Paz	484 (7%)	244 (50%)	240 (50%)
	Sao Paulo	601 (8%)	125 (21%)	476 (79%)
	Fortaleza	313 (4%)	27 (9%)	286 (91%)
	Bogota	1,002 (14%)	292 (29%)	710 (71%)
	Quito	608 (9%)	209 (34%)	399 (66%)
	Lima	653 (9%)	189 (29%)	464 (71%)
	Montevideo	586 (8%)	93 (16%)	493 (84%)
	Caracas	1,029 (15%)	312 (30%)	717 (70%)
	Panama City	311 (4%)	137 (44%)	174 (56%)
	Mexico City	551 (8%)	175 (32%)	376 (68%)

**Table 2:** Partially adjusted associations between high park proximity and neighborhood characteristics with perceived social disorder conditions, based on logistic random intercept models (N = 7,110).

	Social disorder summary (1 or more reported)^i^	Drug use or sale^i i^	Gangs^i i i^	Prostitution^iv^	Assault or crime^v^
	OR (CI)	OR (CI)	OR (CI)	OR (CI)	OR (CI)
*High park proximity*					
Less than 10 min' walk (ref: more than 10 min)	**0.79 (0.71**–**0.88)**	**0.85 (0.77**–**0.94)**	**0.84 (0.76**–**0.93)**	**0.85 (0.74**–**0.98)**	**0.82 (0.74**–**0.91)**

*Neighborhood characteristics*					
Informal neighbourhood (ref: formal neighborhood)	**2.34 (1.95**–**2.81)**	**2.50 (2.13**–**2.93)**	**2.04 (1.75**–**2.36)**	**1.27 (1.05**–**1.54)**	**1.58 (1.37**–**1.84)**

*Street characteristics*					
Home on unpaved street (ref: paved street)	**1.16 (1.01**–**1.35)**	**1.31 (1.15**–**1.50)**	**1.15 (1.00**–**1.30)**	1.04 (0.87–1.26)	**1.15 (1.02**–**1.31)**
Lack of sidewalks (ref: sidewalks present)	1.09 (0.96–1.24)	0.95 (0.85–1.06)	1.09 (0.98–1.23)	0.98 (0.84–1.15)	1.02 (0.90–1.30)
Poor street-lighting (ref: good street-lighting)	**3.93 (3.48**–**4.43)**	**4.79 (4.29**–**5.36)**	**2.30 (2.07**–**2.54)**	**1.63 (1.42**–**1.87)**	**2.28 (2.06**–**2.52)**
Abandoned buildings (ref: no)	**1.95 (1.71**–**2.21)**	**2.16 (1.93**–**2.43)**	**1.48 (1.33**–**1.65)**	**1.30 (1.12**–**1.51)**	**1.32 (1.18**–**1.47)**
Illegal dumping (ref: no)	**3.91 (3.40**–**4.50)**	**4.44 (3.92**–**5.02)**	**2.37 (2.12**–**2.63)**	**1.63 (1.41**–**1.87)**	**2.07 (1.86**–**2.31)**

^i^Adjusted for: sex, length of residency, automobile ownership, having school aged children, education level (high-school or higher), area per person in the household, srh.

^ii^Adjusted for: age, sex, length of neighborhood residency, having school aged children, automobile ownership, area per person in the household, and self-rated health.

^iii^Adjusted for: age, sex, length of neighborhood residency, automobile ownership, education level (high-school or higher), area per person in the household, and self-rated health.

^iv^Adjusted for: age, automobile ownership, employment status (employed vs unemployed), area per person in the household, and self-rated health.

^v^Adjusted for: age, sex, length of neighborhood residency, automobile ownership, area per person in the household, and self-rated health.

Statistically significant coefficients are in bold.

More than half of the sample (54%) reported living in high proximity to a park (less than 10 min’ walk), 18% of the sample resided in informal-neighborhoods and the remaining majority (82%) resided in formal-neighborhoods. Correspondingly, 21% of the participants lived on unpaved streets and 32% had no sidewalks in their residential streets. About half of the sample reported having poor street-lighting (51%), and about a third reported having abandoned buildings (30%) and illegal dumping (33%) three blocks or less from their home. 57% of the participants were female, 63% were employed, but less than a third (31%) owned one (or more) vehicle/s and only 54% had high-school education or higher. Most participants rated their own health as good (65%), only 3% as bad and the remaining 32% as regular. 66% reported visiting parks on a regular basis. On average, participants were 40 years of age, had 24 square meters per person in the household, and have lived in the neighborhood for 20 years. Appendix 3 provides descriptive statistics of the study variables by different social disorder variables.

### 3.2 Associations between perceived social disorder with self-reported park proximity (research question 1) and neighborhood characteristics (research question 2)

As shown in Table 2, compared to participants who reported living more than 10 min’ walk from a park, those who reported having a park in less than 10 min-walk from home were significantly less likely to report each of the four social disorder outcomes (with odds ratios ranging between 0.79 and 0.85). Participants were more likely to report the four social disorder measures if they live in informal neighborhoods, reported having poor street-lighting, abandoned buildings and illegal dumping within three blocks from their home. Participants who did not lived on paved streets were more likely to report drug use/sale and gangs, but not prostitution and assault/crime. Not having sidewalk in participants’ residential street was not associated with any of the social disorder outcomes.

Variables that were found to be significant in the bivariate models (Table 2) were then included in multivariable models to assess the simultaneous impact of self-reported park proximity and neighborhood characteristics on each of the social disorder outcomes (Appendix 1). Interestingly, after adjusting for neighborhood characteristics, high reported park proximity was no longer significantly associated with any of the four social disorder outcomes. Participants were significantly more likely to report at least one type of social disorder if they live in informal neighborhoods and/or in residential streets that were unpaved, poorly lit and with abandoned buildings and illegal dumping. Of the different social disorder domains, drug use or sale was most strongly associated with neighborhood infrastructure followed by gangs, prostitution, and assault/crime. Having poor street-lighting and illegal dumping in participants’ residential streets was significantly associated with all four social disorder outcomes, while living in an informal neighborhood was significantly associated with drug use/sale, gangs, and having abandoned buildings in participants’ residential streets was significantly associated only with higher drug use/sales.

### 3.3 Modification of association between self-reported park proximity and perceived social disorder by neighborhood characteristics (research question 3)

To address the third research question, interaction terms of park proximity and each of the four neighborhood characteristics were added to the multivariable models presented in Appendix 1. Fig. 1 and Appendix 2 present the regression coefficients of the main effects (reported park proximity and each of the neighborhood characteristics) and interaction terms in each of these models. Overall, the odds of reporting at least one social disorder were greater among participants who reported high park proximity, if they live in informal neighborhoods or in poorly lit streets. Of the social disorder outcomes, gangs showed consistent significant associations with all interaction terms suggesting that the odds of perceiving presence of gangs are higher among residents who reported having a park nearby if they also live in an informal neighborhoods or report any of the following conditions in their residential street (within three blocks from home): Poor street-lighting, abandoned buildings, and illegal dumping. Of the neighborhood characteristics, neighborhood informality was found to significantly modify the effect of high park proximity on social disorder for all four social disorder outcomes, suggesting that the odds of perceiving social disorder of any type are higher among those who live in informal neighborhoods and report residing near a park (Fig. 1). In addition, prostitution was more likely to be reported by participants who reported living near a park, if they also reported poor street-lighting in their residential streets.

## 4 Discussion

Despite the potential ambiguous effects of parks on social disorder, serving as both inhibitors and facilitators, only a few recent studies examined this empirically and those were conducted in high-income countries (Boessen and Hipp, 2018, Bogar and Beyer, 2016, Koohsari et al., 2013, Maas et al., 2009). Our study adds to existing knowledge by showing how poor neighborhood infrastructure (e.g., poor street-lighting, presence of abandoned buildings or illegal dumping) is not only associated with increased social disorder but can also modify the associations between park proximity and social disorder. Thus, in areas with high quality and well-maintained infrastructure (e.g., good street-lighting, lack of abandoned buildings and lack of illegal dumping), high park proximity is associated with less social disorder but in areas with poor infrastructure high park proximity is associated with more social disorder.

This study examined the independent and combined associations of self-reported park proximity and neighborhood characteristics (informal neighborhoods, poor street-lighting, presence of abandoned buildings or illegal dumping) with reports of four different social disorders (drug use/sale, gangs, prostitution, assault/crime) in a sample of residents from 11 Latin American cities. Our findings suggest that while having parks near home is associated with less reports of social disorder, this association is precluded in places where neighborhood characteristics and services are poorly developed and/or maintained. Furthermore, participants who reported having a park near home were more likely to report the presence of gangs, if neighborhood characteristics were unfavorable (i.e., informal neighborhoods, poor street-lighting, presence of abandoned buildings or illegal dumping). Those living near a park and residing in informal neighborhoods are more likely to perceive social disorder of any type. Taken together, our results paint a differentiated picture of the role of parks in relation to perceived safety by highlighting the role of contextual factors, such as neighborhood type and neighborhood infrastructure.

Our results make an explicit connection between indicators of social disorder, park proximity, and less favorable neighborhood characteristics. The findings raise important questions about the role of parks in informal neighborhoods, where the presence of public spaces is limited, and population density is high. Neighborhood informality is more than merely an environmental condition, but rather embodies a complex social, economic, and political context, in which these neighborhoods develop and sustain themselves as spatially and socially marginalized communities that are less subject to central regulation through formal surveillance. These circumstances can provide fertile ground for certain behaviors to emerge in common spaces lacking surveillance by a clearly identified group (e.g., park authority, private security, community watch).

Accumulating evidence suggest that parks are unequally distributed within cities around the world (Rigolon et al., 2018, Wolch et al., 2014) and particularly in Latin America (Rigolon et al., 2018, Scopelliti et al., 2016). In our sample, residents of informal neighborhoods reported lower self-reported park proximity compared to those of formal neighborhoods (Appendix 4). However, even when parks are available in informal neighborhoods (as indicated by reports of high park proximity), they are likely to be associated with higher reports of social disorder in those neighborhoods. Although this is precisely the context where public parks are needed to enhance individual mental and physical health, we find that heightened perceptions of social disorders dominate. By contrast, in more privileged neighborhoods perceptions of social disorder were lower (Appendix 4). Thus, neighborhood infrastructure improvements emerge as potential strategies to address intra-urban inequalities. Investing in parks and public spaces and their surrounding built environments, especially in informal neighborhoods, can help mitigate residents’ social disorder perceptions and encourage more parks use thereby allowing residents to enjoy the various health benefits provided by parks.

Comparisons of our findings with prior literature are challenging because only a few studies examined similar interactions and those studies used neighborhood characteristics that are inherently different than those used in our study (Boessen and Hipp, 2018, Koohsari et al., 2013, Maas et al., 2009). For example, in a recent U.S. study, proximity to parks was linked with higher and lower crime in neighborhoods with high and low concentrations of young people, respectively (Boessen & Hipp, 2018). In a study in Hong-Kong, older adults’ concerns about parks' safety were more common among residents of the urban core (characterized by very high density and mixed land uses) compared to residents of a less dense suburban neighborhood (Lo & Jim, 2010). Despite the use of different constructs and measures, these results, along with ours, highlight the importance of neighborhood contextual factors beyond the mere presence of parks, which was also acknowledge recently by scholars (Boessen and Hipp, 2018, Sreetheran and van den Bosch, 2014). Future research is thus needed to further our understanding of the potential combined effect of parks and their surroundings on personal safety, which can then, ultimately, be translated into informative policy guidelines.

Our findings linking reported gang activities with high self-reported park proximity among participants who lived in less favorable environments (according to all neighborhood characteristics), can be explained by territoriality. Previous studies show that gangs tend to operate in parks (Stodolska et al., 2009), which may then be avoided and feared because they are perceived as “belonging” to intimidating groups (Byrne, 2012, Byrne and Wolch, 2009). While in formal neighborhoods, public parks are more likely to be patrolled, fenced and, in some cases, also locked after dark, in informal neighborhoods parks are likely to be open and accessible to all, which, when added to a lack of surveillance, can make them more vulnerable to be occupied by gangs.

Poor street-lighting, reported by half of the sample (see Table 1), was found to be associated with higher reports of social disorder and particularly with the presence of gangs in areas close to parks. It is likely to assume that these increases in social disorder occur mostly after dark, when poor street-lighting becomes especially detrimental causing low visibility and hindering natural surveillance. This, however, cannot be confirmed by our data, which does not distinguish between day and night. Regardless, street-lighting can still reduce social disorder also during the daytime through other mechanisms, for example, by increasing the sense of ownership and community pride (Kamal & Suk, 2018).

A limitation of this study lies in the assessment of the main exposure as park proximity without accounting for parks characteristics, such as parks management, supervised programs, facilities, and amenities. To illustrate, our results suggesting that high park proximity is more strongly related to social disorder in informal compared to formal neighborhoods could be attributed to parks operating hours and lighting. While parks in formal neighborhoods are more likely to be lit and closed to the public after dark, parks in informal neighborhoods are more likely to remain open and lack lighting after dark. Future research should therefore examine the role of parks management and infrastructure in relation to social disorder so as to better support recommendations for increasing personal safety in parks in both high- and low-income areas.

Several other study limitations should also be considered. First, the cross-sectional design allows to determine associations and not causal statements. Similarly, given that this was a secondary data analysis, we were limited to the questions identified a priori in the CAF survey instrument. The survey did not include subpopulations such as children, youth, or older adults for whom parks may play a particularly important role for health and recreation. The use of self-reported data is also subject to biases, such as recall, social desirability, framing biases and/or source-bias (Gullón et al., 2014). For example, it may be that social disorder variables were under-reported in informal neighborhoods, as residents may have refrained from talking about crime out of fear of being viewed or caught as informants. Furthermore, we did not include park characteristics, which are known to be associated with fear of crime (Bedimo-Rung et al., 2005). However, we did control for individual characteristics, which were found to be more influential on perceived safety than environmental factors (Sreetheran & van den Bosch, 2014). Feeling unsafe in general and in association with parks in particular is more likely to be experienced by certain population groups, such as women, children, older-adults, ethnic minorities and low-income individuals (Maas et al., 2009, Sreetheran and van den Bosch, 2014). These groups experience increased fear of crime that is related, but not exclusive, to prior crime victimization, whether direct (e.g., being assaulted, robbed, offered drugs) or indirect (e.g., witnessing crime offenses, learning about crimes reported in the media and/or through interpersonal communication).

A strength of this study lies in its setting in Latin America. By this, our study addresses a research gap that was recently recognized by Sreetheran and Van Den Bosch (2014), who called for more research on associations between crime and parks in developing countries, where crime rates are generally high. Latin American cities especially serve as an intriguing setting for this study, given the paucity of urban green spaces (Rigolon et al., 2018), high crime rates (Sreetheran & Van Den Bosch, 2014), rapid urbanization rates, and high level inequalities (Vereinte Nationen, 2013). Furthermore, the inclusion of informal neighborhoods is noteworthy as the combination of high poverty and poor infrastructure and services make residents of these neighborhoods more susceptible to poor health outcomes (Corburn and Sverdlik, 2019, Smit et al., 2011). Despite these challenges, recent initiatives in Latin America (Sarmiento et al., 2020) underscore the potential of investing in green spaces in informal neighborhoods as a strategy to improve resident’s health. This is particularly important given recent calls (Smit et al., 2011) for more research on health determinants in informal urban settings as part of a larger research and action agenda promoting urban health in low and middle-income countries.

Based on our results we offer several recommendations that can strengthen the potential contribution of parks to community safety and well-being in Latin American cities. As a guiding principle, in addition to infrastructure and programming improvements within parks, a special focus should be given to improvements in areas surrounding parks. Such improvements should aim to prevent, monitor, and address signs of physical disorder, including but not limited to abandoned buildings, limited street-lighting, and illegal dumping. These strategies are critical given that minor physical disorders (e.g., broken windows, vandalism), if unattended, may lead to greater disorder in a vicious cycle (Sampson & Raudenbush, 2004). In addition, it is important to address residents’ sense of safety, which may consequently increase park use, which, on its own, can further enhance community safety in a virtuous cycle. Having supervised programs and activities in parks was identified in South America (Sarmiento et al., 2017) and elsewhere (Bedimo-Rung et al., 2005, Cohen et al., 2010) as an effective strategy to draw community members to visit parks. The availability of such programs in parks can attract more park users and thereby strengthen natural surveillance (i.e., self-surveillance by users, passers-by, and residents) which will increase perceived safety. While these strategies may be broadly applicable, concentrated efforts should be done to prioritize areas near parks in informal neighborhood and in areas with deficient infrastructure (unpaved streets, poor street-lighting etc.), where perceived social disorder was found to be higher than elsewhere. Such focal interventions may maximize the safety and health benefits provided by existing parks to their surroundings, and ultimately also help mitigate intra-urban inequalities in personal safety and well-being.

## Funding body

*“The Salud Urbana en América Latina (SALURBAL)/ Urban Health in Latin America project is funded by the Wellcome Trust [205177/Z/16/Z]. For the purpose of open access, the author has applied a CC-BY public copyright license to any Author Accepted Manuscript version of this publication.”*

## Declaration of Competing Interest

The authors declare that they have no known competing financial interests or personal relationships that could have appeared to influence the work reported in this paper.
