# Supplementary material for: Is self-reported park proximity associated with perceived social disorder? Findings from eleven cities in Latin America
Source: Landsc Urban Plan. 2022 Mar;219:None. doi: 10.1016/j.landurbplan.2021.104320 (PMC8780619; doi:10.1016/j.landurbplan.2021.104320)
Supplement: Supplementary data 1 [file mmc1.docx]

**Appendix 1:**  Fully adjusted models of associations between perceived social disorder, high park proximity, and neighborhood characteristics (N=7,110)

|  | **Social disorder summary**  **(1 or more reported)ⁱ** | **Drug use/saleⁱⁱ** | **Gangsⁱⁱⁱ** | **Prostitutionⁱᵛ** | **Assault or crimeᵛ** |
| --- | --- | --- | --- | --- | --- |
|  | **OR (CI)** | **OR (CI)** | **OR (CI)** | **OR (CI)** | **OR (CI)** |
| ***High park proximity*** |  |  |  |  |  |
| Less than 10 minutes' walk  (ref: more than 10 min) | 0.93 (0.82-1.05) | 1.06 (0.94-1.18) | 0.96 (0.86-1.06) | 0.92 (0.80-1.06) | 0.91 (0.82-1.01) |
| ***Neighborhood characteristics*** |  |  |  |  |  |
| Informal neighborhood | **1.38 (0.82-1.69)** | **1.46 (1.22-1.74)** | **1.51 (1.29-1.77)** | 1.04 (0.85-1.27) | 1.16 (0.99-1.36) |
| ***Street characteristics*** |  |  |  |  |  |
| Home on unpaved street (ref: paved street) | **0.78 (0.66-0.91)** | **0.82 (0.71-0.96)** | **0.87 (0.76-0.99)** | NI | 0.91 (0.73-1.04) |
| Lack of sidewalks (ref: sidewalks present) | NI | NI | NI | NI | NI |
| Poor street-lighting (ref: good street-lighting) | **2.88 (2.53-3.27)** | **3.43 (3.04-3.86)** | **1.82 (1.63-2.03)** | **1.42 (1.23-1.65)** | **1.93 (1.73-2.16)** |
| Abandoned buildings (ref: no) | **1.28 (1.12-1.47)** | **1.38 (1.21-1.56)** | 1.11 (0.99-1.25) | 1.11 (0.95-1.29) | 1.00 (0.90-1.13) |
| Illegal dumping (ref: no) | **2.47 (2.12-2.88)** | **2.68 (2.34-3.07)** | **1.76 (1.56-1.98)** | **1.37 (1.17-1.60)** | **1.58 (1.41-1.78)** |
| *Constant* | 2.67 (1.54-4.62) | 0.86 (0.45-1.64) | 1.76 (1.56-1.98) | **0.16 (0.08-0.30)** | 1.46 (0.94-2.29) |
| *Variance of random intercept* | 0.52 (0.22-1.24) | **0.70 (0.30-0.64)** | **0.12 (0.05-0.31)** | **0.47 (0.20-0.72)** | **0.20 (0.08-0.50)** |
| *Number of observations* | 7,110 | 7,110 | 7,110 | 7,110 | 7,110 |
| *Number of groups* | 11 | 11 | 11 | 11 | 11 |
| *AIC* | 7,400.975 | 8,101.248 | 9,091.015 | 5,916.99 | 9,284.81 |

ⁱ Adjusted for: sex, length of residency, automobile ownership, having school aged children, education level (high-school or higher), area per person in the household, srh.

ⁱⁱ Adjusted for: age, sex, length of neighborhood residency, having school aged children, automobile ownership, area per person in the household, and self-rated health.

**ⁱⁱⁱ** Adjusted for: age, sex, length of neighborhood residency, automobile ownership, education level (high-school or higher), area per person in the household, and self-rated health.

ⁱᵛAdjusted for: age, automobile ownership, employment status (employed vs unemployed), area per person in the household, and self-rated health.

ᵛ Adjusted for: age, sex, length of neighborhood residency, automobile ownership, area per person in the household, and self-rated health.

**Statistically significant coefficients are in bold**, NI = Not Included
