# Supplementary material for: Is self-reported park proximity associated with perceived social disorder? Findings from eleven cities in Latin America
Source: Landsc Urban Plan. 2022 Mar;219:None. doi: 10.1016/j.landurbplan.2021.104320 (PMC8780619; doi:10.1016/j.landurbplan.2021.104320)
Supplement: Supplementary data 2 [file mmc2.docx]

| **Appendix 2:** Main and interaction effects of high park proximity and neighborhood characteristics on perceived social disorder (n=7,110) | | | | | |  |
| --- | --- | --- | --- | --- | --- | --- |
|  | **Social disorder summary**  **(1 or more reported) ⁱ** | **Drug use or sale ⁱⁱ** | **Gangs ⁱⁱⁱ** | **Prostitution ⁱᵛ** | **Assault or crime ᵛ** |  |
|  | **OR (CI)** | **OR (CI)** | **OR (CI)** | **OR (CI)** | **OR (CI)** |  |
| ***Park Proximity X Informal neighborhood*** |  |  |  |  |  |  |
| High park proximity - less than 10 minutes' walk  (ref: more than 10 min) | **0.86 (0.76-0.98)*** | 1.006 (0.89-1.14) | **0.86 (0.77-.97)*** | **0.83 (0.71-0.97)*** | **0.86 (0.77-0.96)**** |  |
| Informal neighborhood  (ref: formal neighborhood) | 1.12 (-0.87-1.43) | **1.26 (1.009-1.57)*** | 1.15 (0.95-1.40) | 0.83 (0.65-1.07) | 1.00 (0.82-1.22) |  |
| High park proximity X Informal neighborhood | **1.66 (1.17-2.35)**** | **1.39 (1.02-1.89)*** | **1.81 (1.39-2.36)**** | **1.65 (1.19-2.29)**** | **1.38 (1.06-1.79)*** |  |
| ***Park Proximity X Poor street-lighting*** |  |  |  |  |  |  |
| High park proximity - less than 10 minutes' walk  (ref: more than 10 min) | **0.82 (0.71-0.96)*** | 1.02 (0.88-1.18) | **0.85 (0.73-0.98)*** | **0.77 (0.63-0.95)*** | **0.83 (0.72-0.96)*** |  |
| Poor streetlights  (ref: good street-lighting) | **2.41 (2.01-2.89)***** | **3.28 (2.77-3.89)***** | **1.59 (1.36-1.86)***** | 1.20 (0.98-1.48) | **1.76 (1.50-2.05)***** |  |
| High park proximity X Poor street-lighting | **1.38 (1.09-1.75)**** | 1.08 (0.87-1.35) | **1.28 (1.04-1.56)*** | **1.36 (1.04-1.77)*** | 1.19 (0.97-1.45) |  |
| ***Park Proximity X Abandoned buildings*** |  |  |  |  |  |  |
| High park proximity - less than 10 minutes' walk  (ref: more than 10 min) | **0.86 (0.75-0.99)*** | 1.04 (0.91-1.19) | **0.84 (0.75-0.95)**** | 0.88 (0.75-1.04) | **0.87 (0.77-0.99)*** |  |
| Abandoned buildings  (ref: no) | **1.30 (1.07-1.58)**** | **1.34 (1.11-1.60)**** | 0.88 (0.75-1.04) | 1.03 (0.83-1.27) | 0.93 (0.79-1.10) |  |
| High park proximity X Abandoned buildings | 1.26 (0.97-1.63) | 1.06 (0.83-1.34) | **1.54 (1.24-1.91)***** | 1.16 (0.87-1.55) | 1.15 (0.93-1.42) |  |
| ***Park Proximity X Illegal dumping*** |  |  |  |  |  |  |
| High park proximity - less than 10 minutes' walk  (ref: more than 10 min) | 0.89 (0.78-1.02) | 1.06 (0.93-1.21) | **0.86 (0.76-0.98)*** | 0.92 (0.77-1.10) | 0.91 (0.80-1.03) |  |
| Illegal dumping  (ref: no) | **3.23 (2.65-3.93)***** | **2.70 (2.25-3.24)***** | **1.50 (1.28-1.76)***** | **1.37 (1.11-1.69)**** | **1.59 (1.35-1.87)***** |  |
| High park proximity X Illegal dumping | 1.08 (0.82-1.42) | 0.93 (0.77-1.26) | **1.37 (1.11-1.69)**** | 1.001 (0.76-1.32) | 0.99 (0.80-1.22) |  |
| ⁱ Adjusted for: sex, length of residency, automobile ownership, having school aged children, education level (high-school or higher), area per person in the household, srh.  ⁱⁱ Adjusted for: age, sex, length of neighborhood residency, having school aged children, automobile ownership, area per person in the household, and self-rated health.  **ⁱⁱⁱ** Adjusted for: age, sex, length of neighborhood residency, automobile ownership, education level (high-school or higher), area per person in the household, and self-rated health.  ⁱᵛAdjusted for: age, automobile ownership, employment status (employed vs unemployed), area per person in the household, and self-rated health.  ᵛ Adjusted for: age, sex, length of neighborhood residency, automobile ownership, area per person in the household, and self-rated health.  * p ≤ .05 ; ** p ≤ .01 ; *** p ≤ .001; **Significant coefficients are in bold** | | | | | | |
|  | | | | | | |
