# Supplementary material for: Is self-reported park proximity associated with perceived social disorder? Findings from eleven cities in Latin America
Source: Landsc Urban Plan. 2022 Mar;219:None. doi: 10.1016/j.landurbplan.2021.104320 (PMC8780619; doi:10.1016/j.landurbplan.2021.104320)
Supplement: Supplementary data 3 [file mmc3.docx]

| Appendix 3: Descriptive statistics for the study variables in the total sample and by different social disorder variables | | | | | | | | | | |
| --- | --- | --- | --- | --- | --- | --- | --- | --- | --- | --- |
| Variables | | Total sample (n=7,110**)** | Drug use/sales | | Gangs | | Prostitution | | Assault/crime | |
|  |  |  | **No (n=3,078)** | **Yes (n=4,032)** | **No (n=3,968)** | **Yes (n=3,142)** | **No (n=5,958)** | **Yes (n=1,152)** | **No (n=3,649)** | **Yes (n=3,461)** |
| *Neighborhood social disorder* | Drug use/sales | 4,032  (57%) | 0  (0%) | 4,032 (100%) | 1,687 (42%) | 2,345 (58%) | 3,141 (78%) | 891  (22%) | 1,531 (38%) | 2,501 (62%) |
|  | Gangs | 3,142  (44%) | 797 (25%) | 2,345 (75%) | 0  (0%) | 3,142 (100%) | 2,309 (73%) | 833  (27%) | 861 (27%) | 2,281 (73%) |
|  | Prostitution | 1,152  (16%) | 261 (23%) | 891 (77%) | 319 (28%) | 833  (72%) | 0  (0%) | 1,152 (100%) | 256 (22%) | 896  (78%) |
|  | Assault or crime | 3,461  (49%) | 960 (28%) | 2,501 (72%) | 1,180 (34%) | 2,281 (66%) | 2.565 (74%) | 896  (26%) | 0  (0%) | 3,461 (100%) |
| *Park proximity* | |  |  |  |  |  |  |  |  |  |
|  | Less than 10 minutes' walk | 3,867  (54%) | 1,694 (44%) | 2,173 (56%) | 2,214 (57%) | 1,653 (43%) | 3,288 (85%) | 579  (15%) | 2,040 (53%) | 1,827 (47%) |
|  | More than 10 minutes’ walk | 3,243  (46%) | 1,384 (43%) | 1,859 (57%) | 1,754 (54%) | 1,489  (46%) | 2,670 (82%) | 573  (18%) | 1,609 (50%) | 1,634  (50%) |
| *Neighborhood characteristics* | |  |  |  |  |  |  |  |  |  |
| *Neighborhood type* | Informal | 1,294  (18%) | 331 (26%) | 963 (74%) | 502 (39%) | 792  (61%) | 1,035 (80%) | 259  (20%) | 509 (39%) | 785  (61%) |
|  | Formal | 5,816  (82%) | 2,747 (47%) | 3,069 (53%) | 3,466 (60%) | 2,350 (40%) | 4,923 (85%) | 893  (15%) | 3,140 (54%) | 2,676 (46%) |
| *Street characteristics* | |  |  |  |  |  |  |  |  |  |
| *Street pavement* | House on other street type (i.e., dirt, alleyway, other) | 1,471  (21%) | 599 (41%) | 872 (59%) | 725 (49%) | 746  (51%) | 1,279 (87%) | 192  (13%) | 730 (50%) | 741  (50%) |
|  | House on paved street | 5,639  (79%) | 2,479 (44%) | 3,160 (56%) | 3,243 (58%) | 2,396 (42%) | 4,679 (83%) | 960  (17%) | 2,919 (52%) | 2,720 (48%) |
| *Sidewalk* | No sidewalks in residential street (within block) | 2,242  (32%) | 1,025 (46%) | 1,217 (54%) | 1,255 (56%) | 987  (44%) | 1,852 (83%) | 390  (17%) | 1,174 (52%) | 1,068 (48%) |
|  | Sidewalks are present in residential street (within block) | 4,868  (68%) | 2,053 (42%) | 2,815 (58%) | 2,713 (56%) | 2,155 (44%) | 4,106 (84%) | 762  (16%) | 2,475 (51%) | 2,393 (49%) |
| *Street-lighting* | Poor street-lighting within three blocks | 3,609  (51%) | 966 (28%) | 2,535 (72%) | 1,585 (45%) | 1,916 (55%) | 2,819 (81%) | 682  (19%) | 1,451 (41%) | 2,050 (59%) |
|  | Good street-lighting within three blocks | 3,501  (49%) | 2,112 (59%) | 1,497 (41%) | 2,383 (66%) | 1,226 (34%) | 3,139 (87%) | 470  (13%) | 2,198 (61%) | 1,411 (39%) |
| *Abandoned building* | There are abandoned buildings within three blocks | 2,112  (30%) | 670 (32%) | 1,442 (68%) | 1,045 (49%) | 1,067 (51%) | 1,768 (84%) | 344  (16%) | 1,005 (48%) | 1,107 (52%) |
|  | There are no abandoned buildings within three blocks | 4,998  (70%) | 2,408 (48%) | 2,590 (52%) | 2,923 (58%) | 2,075 (42%) | 4,190 (84%) | 808  (16%) | 2,644 (53%) | 2,354 (47%) |
|  |  |  |  |  |  |  |  |  |  |  |

| Variables | | Total sample (n=7,110) | Drug use/sales | | Gangs | | Prostitution | | Assault/crime | |
| --- | --- | --- | --- | --- | --- | --- | --- | --- | --- | --- |
|  |  |  | **No (n=3,078)** | **Yes (n=4,032)** | **No (n=3,968)** | **Yes (n=3,142)** | **No (n=5,958)** | **Yes (n=1,152)** | **No (n=3,078)** | **Yes (n=4,032)** |
| *Waste dumping* | There is illegal dumping within three blocks | 2,340  (33%) | 583  (25%) | 1,757 (75%) | 971  (42%) | 1,369 (58%) | 1,856 (79%) | 484  (21%) | 929  (40%) | 1,411 (60%) |
|  | There is no illegal dumping within three blocks | 4,770  (67%) | 2,495 (52%) | 2,275 (48%) | 2,997 (63%) | 1,773 (37%) | 4,102 (86%) | 668  (14%) | 2,720 (57%) | 2,050 (43%) |
| *Individual characteristics* | |  |  |  |  |  |  |  |  |  |
| *Sex* | Male (1) | 3,038  (43%) | 1,395 (46%) | 1,643 (54%) | 2,214 (54%) | 1,858 (46%) | 3,425 (84%) | 647  (16%) | 2,000 (49%) | 2,072 (51%) |
|  | Female (0) | 4,072  (57%) | 1,683 (41%) | 2,389 (59%) | 1,754 (58%) | 1,284 (42%) | 2,533 (83%) | 505  (17%) | 1,649 (54%) | 1,389 (46%) |
|  | Age [M(SD)] | 40.10  (0.14) | 40.32 (0.20) | 39.92 (0.18) | 40.25 (0.17) | 39.89 (0.20) | 39.98 (0.14) | 40.70 (0.32) | 40.35 (0.18) | 39.83 (0.19) |
|  | Length of neighborhood residency in years [M(SD)] | 20.30  (0.18) | 19.94 (0.28) | 20.57 (0.23) | 19.95 (0.24) | 20.74 (0.26) | 20.05 (0.19) | 21.56 (0.44) | 19.98 (0.26) | 20.63 (0.24) |
| *School aged children* | Have school aged children | 4,604  (65%) | 1,936 (42%) | 2,668 (58%) | 2,548 (55%) | 2,056 (45%) | 3,852 (84%) | 752  (16%) | 2,318 (50%) | 2,286 (50%) |
|  | Does not have school aged children | 2,506  (35%) | 1,142 (46%) | 1,364 (54%) | 1,420 (57%) | 1,086 (43%) | 2,106 (84%) | 400  (16%) | 1,331 (53%) | 1,175 (47%) |
| *Parks use* | Park user | 4,690  (66%) | 2,081 (44%) | 2,609 (56%) | 2,617 (56%) | 2,073 (44%) | 3,968 (85%) | 722  (15%) | 2,441 (52%) | 2,249 (48%) |
|  | Non-park user | 2,420  (34%) | 997  (41%) | 1,423 (59%) | 1,351 (56%) | 1,069 (44%) | 1,990 (82%) | 430  (18%) | 1,208 (50%) | 1,212 (50%) |
| *Automobile ownership* | Automobile owner | 2,235  (31%) | 1,082 (48%) | 1,153 (52%) | 1,401 (63%) | 834  (37%) | 1,909 (85%) | 326  (15%) | 1,259 (56%) | 976  (44%) |
|  | Non-automobile owner | 4,875  (69%) | 1,996 (41%) | 2,879 (59%) | 2,567 (53%) | 2,308 (47%) | 4,049 (83%) | 826  (17%) | 2,390 (49%) | 2,485 (51%) |
| *Employment status* | Employed | 4,548  (64%) | 2,008 (44%) | 2,540 (56%) | 2,556 (56%) | 1,992 (44%) | 3,780 (83%) | 768  (17%) | 2,361 (52%) | 2,187 (48%) |
|  | Unemployed | 2,562  (36%) | 1,070 (42%) | 1,492 (58%) | 1,412 (55%) | 1,150 (45%) | 3,780 (83%) | 768  (17%) | 1,288 (50%) | 1,274 (50%) |
|  | Overcrowding - Area per person in the household (m²) [M(SD)] | 23.88  (0.23) | 25.77 (0.37) | 22.43 (0.30) | 25.19 (0.33) | 22.22 (0.32) | 24.12 (0.26) | 22.62 (0.52) | 25.18 (0.34) | 22.50 (0.32) |
| *Education* | Less than high school | 3,270  (46%) | 1,242 (40%) | 2,028 (60%) | 1,729 (53%) | 1,541 (47%) | 2,711 (82%) | 559  (18%) | 1,593 (49%) | 1,677 (51%) |
|  | High school or higher | 3,840  (54%) | 1,836 (60%) | 2,004 (40%) | 2,239 (58%) | 1,601 (42%) | 3,247 (84%) | 593  (16%) | 2,056 (54%) | 1,784 (46%) |
| *Self-rate health* | Bad | 231  (3%) | 77  (33%) | 154  (67%) | 115  (50%) | 116  (50%) | 187  (81%) | 44  (19%) | 105  (45%) | 126  (55%) |
|  | Regular | 2,274  (32%) | 965  (42%) | 1,309 (58%) | 1,179 (52%) | 1,095 (48%) | 1,889 (83%) | 385  (17%) | 1,103 (49%) | 1,171 (51%) |
|  | Good | 4,605  (65%) | 2,036 (44%) | 2,569 (56%) | 2,674 (58%) | 1,931 (42%) | 3,882 (84%) | 723  (16%) | 2,441 (53%) | 2,164 (47%) |

| Variables | | Total sample (n=7,110) | Drug use/sales | | Gangs | | Prostitution | | Assault/crime | |
| --- | --- | --- | --- | --- | --- | --- | --- | --- | --- | --- |
|  |  |  | **No (n=3,078)** | **Yes (n=4,032)** | **No (n=3,968)** | **Yes (n=3,142)** | **No (n=5,958)** | **Yes (n=1,152)** | **No (n=3,078)** | **Yes (n=4,032)** |
| *City of residence* | |  |  |  |  |  |  |  |  |  |
|  | Buenos Aires | 972  (14%) | 325  (33%) | 647  (67%) | 491  (51%) | 481  (49%) | 832  (86%) | 140  (14%) | 428  (44%) | 544  (56%) |
|  | La Paz | 484  (7%) | 365  (75%) | 119  (25%) | 278  (57%) | 206  (43%) | 435  (90%) | 49  (10%) | 311  (64%) | 173  (36%) |
|  | Sao Paulo | 601  (8%) | 223  (37%) | 378  (63%) | 458  (76%) | 143  (24%) | 482  (80%) | 119  (20%) | 307  (51%) | 294  (49%) |
|  | Fortaleza | 313  (4%) | 58  (19%) | 255  (82%) | 132  (42%) | 181  (58%) | 185  (59%) | 128  (41%) | 89  (28%) | 224  (72%) |
|  | Bogota | 1,002  (14%) | 411  (41%) | 591  (59%) | 507  (51%) | 495  (49%) | 933  (93%) | 69  (7%) | 543  (54%) | 459  (46%) |
|  | Quito | 608  (9%) | 292  (48%) | 316  (52%) | 305  (50%) | 303  (50%) | 574  (94%) | 34  (6%) | 375  (62%) | 233  (38%) |
|  | Lima | 653  (9%) | 267  (41%) | 386  (59%) | 323  (49%) | 330  (51%) | 578  (89%) | 75  (11%) | 328  (50%) | 325  (50%) |
|  | Montevideo | 586  (8%) | 177  (30%) | 409  (70%) | 381  (65%) | 205  (35%) | 481  (82%) | 105  (18%) | 246  (42%) | 340  (58%) |
|  | Caracas | 1,029  (14%) | 496  (48%) | 533  (52%) | 556  (54%) | 473  (46%) | 757  (74%) | 272  (26%) | 498  (48%) | 531  (52%) |
|  | Panama City | 311  (4%) | 192  (62%) | 119  (38%) | 214  (69%) | 97  (31%) | 265  (85%) | 46  (15%) | 203  (65%) | 108  (35%) |
|  | Mexico City | 551  (8%) | 272  (49%) | 279  (51%) | 323  (59%) | 228  (41%) | 436  (79%) | 115  (21%) | 321  (58%) | 230  (42%) |
