# Supplementary material for: Is self-reported park proximity associated with perceived social disorder? Findings from eleven cities in Latin America
Source: Landsc Urban Plan. 2022 Mar;219:None. doi: 10.1016/j.landurbplan.2021.104320 (PMC8780619; doi:10.1016/j.landurbplan.2021.104320)
Supplement: Supplementary data 4 [file mmc4.docx]

| Appendix 4: Comparison between formal and informal neighborhoods | | | | |
| --- | --- | --- | --- | --- |
| Variables |  | **Formal neighborhoods (n=5,816)** | **Informal neighborhoods**  **(n=1,294)** | **χ²/T** |
| *Social disorder* | |  |  |  |
| Social disorder summary (one or more) | Yes | 3,994 (69%) | 1,087 (98%) | **121.99** |
|  | No | 1,822 (31%) | 207 (2%) |  |
| Drug use/sale | Yes | 3,069 (53%) | 963 (75%) | **202.14** |
|  | No | 2,747 (47%) | 331 (25%) |  |
| Gangs | Yes | 2,350 (40%) | 792 (61%) | **185.68** |
|  | No | 3,466 (60%) | 502 (39%) |  |
| Prostitution | Yes | 893 (15%) | 259 (20%) | **16.94** |
|  | No | 4,923 (85%) | 1,035 (80%) |  |
| Assault or crime | Yes | 2,676 (46%) | 785 (61%) | **90.98** |
|  | No | 3,140 (54%) | 509 (39%) |  |
| *Park proximity* | |  |  |  |
|  | Less than 10 minutes' walk | 3,305 (57%) | 562 (43%) | **183.60** |
|  | More than 10 minutes’ walk | 2,511 (43%) | 732 (57%) |  |
| *Street characteristics* | |  |  |  |
| *Street pavement* | Unpaved street within block (i.e., dirt, alley) | 960 (17%) | 511 (39%) | **340.77** |
|  | Paved street within block | 4,856 (83%) | 783 (61%) |  |
| *Sidewalk* | No sidewalks in residential street | 1,626 (28%) | 616 (48%) | **189.25** |
|  | Sidewalks are present in residential street | 4,190 (72%) | 678 (52%) |  |
| *Street-lighting* | Poor street-lighting within three blocks | 2,565 (44%) | 936 (72%) | **337.53** |
|  | Good street-lighting within three blocks | 3,251 (56%) | 358 (28%) |  |
| *Abandoned building* | There are Abandoned buildings within three blocks | 1,701 (29%) | 411 (32%) | 3.21 |
|  | There are No abandoned buildings within three blocks | 4,115 (71%) | 883 (68%) |  |
| *Waste dumping* | There are Illegal dumping within three blocks | 1,579 (27%) | 761 (59%) | **480.54** |
|  | There are No illegal dumping within three blocks | 4,237 (73%) | 533 (41%) |  |
| *Individual characteristics* | | | | |
| *Sex* | Male (1) | 2,534 (44%) | 504 (39%) | **9.23** |
|  | Female (0) | 3,282 (56%) | 790 (61%) |  |
|  | Age [M(SD)]* | 40.23 (0.14) | 39.50 (0.32) | **2.11** |
|  | Length of neighborhood residency in years [M(SD)] | 20.17 (0.20) | 20.85 (0.39) | -1.47 |

| Variables |  | Formal neighborhoods (n=5,816) | Informal neighborhoods  (n=1,294) |  |
| --- | --- | --- | --- | --- |
| *School aged children* | Have school aged children | 3,742 (64%) | 862 (64%) | 2.40 |
|  | Does not have school aged children | 2,074 (36%) | 432 (36%) |  |
| *Parks use* | Park user | 4,021 (69%) | 669 (52%) | **143.34** |
|  | Non-park user | 1,795 (31%) | 625 (48%) |  |
| *Automobile ownership* | Automobile owner | 2,071 (36%) | 1,130 (87%) | **258.32** |
|  | Non-automobile owner | 3,756 (64%) | 164 (13%) |  |
| *Employment status* | Employed | 3,760 (65%) | 788 (61%) | **6.47** |
|  | Unemployed | 2,056 (35%) | 506 (39%) |  |
|  | Overcrowding - Area per person in the household (m²) [M(SD)] | 25.77 (0.26) | 15.37 (0.41) | **17.59** |
| *Education* | Less than high school | 2,417 (42%) | 853 (66%) | **252.91** |
|  | High school or higher | 3,399 (58%) | 441 (34%) |  |
| *Self-rate health* | Bad | 189 (3%) | 42 (3%) | **38.06** |
|  | Regular | 1,953 (34%) | 321 (25%) |  |
|  | Good | 3,674 (63%) | 931 (72%) |  |

**Significant values are in bold**
